# Discrimination of *Klebsiella pneumoniae* and *Klebsiella quasipneumoniae* by MALDI‐TOF Mass Spectrometry Coupled With Machine Learning

**DOI:** 10.1002/mbo3.70035

**Published:** 2025-07-15

**Authors:** Mari Nishikawa, Wenhao Tang, Markus Kostrzewa, Jonah Rodgus, Frances Davies, Yi Liu, Elita Jauneikaite, Gerald Larrouy‐Maumus

**Affiliations:** ^1^ Faculty of Natural Sciences, Department of Life Sciences, Centre for Bacterial Resistance Biology Imperial College London UK; ^2^ Faculty of Natural Sciences, Department of Mathematics Imperial College London London UK; ^3^ Bruker Daltonics GmbH&Co. KG Bremen Germany; ^4^ NIHR Health Protection Research Unit in Healthcare Associated Infections and Antimicrobial Resistance, Department of Infectious Disease Imperial College London London UK; ^5^ Imperial College Healthcare NHS Trust London UK; ^6^ Department of Microbiology North West London Pathology London UK

**Keywords:** identification, *Klebsiella*, lipids, MALDI

## Abstract

*Klebsiella* species, including *Klebsiella pneumoniae* and *Klebsiella quasipneumoniae*, present significant challenges in clinical microbiology due to their genetic similarity, which complicates accurate species identification using established methods, including matrix‐assisted laser desorption/ionization‐time of flight mass spectrometry (MALDI‐TOF MS) on the protein/peptide level. Although the treatment choice for infections caused by these pathogens is often similar, precise species characterization enhances our epidemiological understanding. While whole‐genome sequencing can accurately distinguish *Klebsiella* species accurately, those analyses are time‐consuming, requiring specialized expertise, and are not currently used in routine clinical laboratories. Therefore, developing a timely and accurate pathogen characterization method is essential for effective treatment, management, and infection control measures. This study combined MALDI‐TOF MS in negative ion mode with machine learning techniques to identify potential lipid biomarkers as a novel method to distinguish between *K. pneumoniae* and *K. quasipneumoniae*. Using this method, we identified discriminative features between the species, with peaks at *m/z* 2157, *m/z* 1931, *m/z* 1964, *m/z* 2042, and *m/z* 1407 highlighted as potential biomarkers for species identification. Our findings suggest that the lipid profiles of the species obtained from MALDI‐TOF MS can serve as effective biomarkers for distinguishing *Klebsiella* species. Further research should focus on the structural identification of these biomarkers and expand the data set to include more isolates for each of the species. This approach holds promise for developing more cost‐effective and rapid diagnostic tools in clinical microbiology, ultimately improving patient outcomes and infection control.

## Graphical abstract

Lipid profiling using routine MALDI‐TOF MS can serve as effective biomarkers for distinguishing *Klebsiella* species.

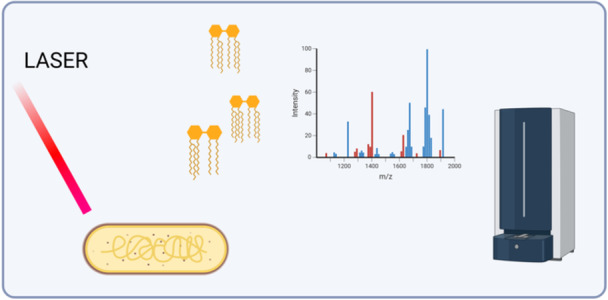

## 1 Introduction

*Klebsiella* species are common Gram‐negative bacteria with complex population structures. *Klebsiella pneumoniae* is a major cause of nosocomial infections, including bloodstream infections, urinary tract infections, and pneumonia. Infections are especially prevalent among immunocompromised or critically ill patients, such as those in intensive care units (Hafiz et al. 2023; Choby et al. 2020).

In 2014, whole‐genome sequencing has identified two new species, namely *Klebsiella quasipneumoniae* and *Klebsiella variicola*, from isolates that were originally classified as *K. pneumoniae* (Holt et al. 2015; Long et al. 2017). Being highly genetically similar to *K. pneumoniae*, *K. quasipneumoniae* isolates are often misidentified as *K. pneumoniae*; indeed, it is hard to distinguish the two species using routine laboratory techniques (Long et al. 2017; Martinez‐Romero et al. 2018). Although whole‐genome sequencing provides accurate species identification, its time‐consuming nature and requirement for expertise limit its routine use in diagnostic laboratories. Multiplex polymerase chain reaction (PCR) can distinguish these species using chromosomally encoded genes (*bla*_SHV_, *bla*_LEN_ and *bla*_OKP_) and their flanking gene (*deoR*) (Fonseca et al. 2017); however, its sensitivity is limited especially when detecting low levels of bacterial DNA in clinical samples. Nevertheless, since *Klebsiella* species isolates can possess different virulence and antibiotic resistance profiles (Holt et al. 2015), accurate species identification is essential for our improved understanding of the epidemiological and clinical trends of these two species.

Matrix‐assisted laser desorption/ionization time‐of‐flight mass spectrometry (MALDI‐TOF MS) is an analytical technique widely used in biochemistry and molecular biology for rapid and accurate determination of molecular masses of biomolecules, such as proteins, peptides, nucleic acids, and carbohydrates, which can be used for species identification. While ribosomal proteins serve as common biomarkers in conventional MALDI‐TOF MS, they lack sufficient resolution for closely related species like *Klebsiella*, leading to species misclassification (Long et al. 2017; Solntceva et al. 2020; Dinkelacker et al. 2018). An alternative approach targets lipid fingerprints, specifically lipid A—the hydrophobic anchor of lipopolysaccharides in the outer membrane of Gram‐negative bacteria (Solntceva et al. 2020; Furniss et al. 2020). The diverse modification patterns of lipid A can be regulated by environmental conditions, such as pH (Raetz and Whitfield 2002; Saha et al. 2022; Mozaheb et al. 2023). These heterogeneous lipid fingerprints represent promising biomarkers for precise species identification for *Klebsiella* species, which is crucial for effective infection management and control.

This study aims to accelerate the application of routine MALDI‐TOF MS with lipid profiling to address the challenge of rapid discrimination between *K. pneumoniae* and *K. quasipneumoniae*. Analysis using MALDI‐TOF MS in the negative‐ion mode coupled with a machine learning algorithm demonstrated the feasibility of distinguishing *K. pneumoniae* and K. *quasipneumoniae* based on their lipid profiles.

## 2 Material and Methods

### 2.1 Bacterial Strains and Species Confirmation From Whole‐Genome Sequencing Data

A total of 39 bacterial blood culture isolates, including 20 *K. pneumoniae* and 19 *K. quasipneumoniae* isolates, were used in this study (Table S1). For genomic DNA preparation, all bacterial isolates were grown from frozen stocks onto LB agar (Sigma‐Aldrich, Milwaukee, USA) at 37°C overnight. Overnight growth was resuspended in PBS (Sigma‐Aldrich, Milwaukee, USA) and processed following the instructions provided by the MicrobesNG (Birmingham, UK). Then the prepared bacterial lysates were sent to MicrobesNG (https://microbesng.com) for whole‐genome sequencing. Genomic DNA libraries were prepared using the Nextera XT Library Prep Kit (Illumina, San Diego, USA) following the manufacturer's protocol with the following modifications: input DNA is increased twofold, and PCR elongation time is increased to 45 s. DNA quantification and library preparation were carried out on a Hamilton Microlab STAR automated liquid handling system (Hamilton Bonaduz AG, Switzerland). Libraries were sequenced on an Illumina NovaSeq. 6000 (Illumina, San Diego, USA) using a 250 bp paired end protocol.

FastQC v0.12.1 (https://www.bioinformatics.babraham.ac.uk/projects/fastqc/) and MultiQC v0.4 (https://github.com/MultiQC/MultiQC) were used to assess the quality of raw sequence reads. Trimmomatic v0.39 (Bolger et al. 2014) was used to trim raw sequence reads. Reads were trimmed per specific instructions: to remove leading low quality or N bases (below quality three); to remove trailing low quality or N bases (below quality three); to scan the read with a 10‐base wide sliding window, cutting when the average quality per base drops below 30, and to drop reads below 50 bases long. Bacterial species were confirmed from trimmed reads using Kraken2 v2.0.7‐beta (Wood et al. 2019) and Bracken v2.5.0 (https://github.com/jenniferlu717/Bracken) with the standard bacterial Kraken2 database (last updated September 15, 2023).

### 2.2 Sample Preparation for Lipid Profiling

Bacterial strains were cultured in LB medium overnight at 37°C. Bacteria were then pelleted, heat‐inactivated (98°C for 1 h), and washed three times with 200 µL ddH_2_O. Acid hydrolysis of the bacteria was performed using 1% acetic acid, followed by incubation at 98°C for 2 h. The bacteria were then washed three more times with 200 µL ddH_2_O and resuspended in 200 µL ddH_2_O. For MALDI‐TOF MS analysis, 0.5 µL of the sample and 0.5 µL MBT lipid Xtract matrix (Bruker) were loaded on a MALDI target plate named MSP 96 target polished steel BC (Bruker Part‐No. 8280800). The bacterial suspension and matrix were mixed directly on the target by pipetting and then dried gently under a stream of air. This procedure was conducted on three technical replicates of 39 samples comprising a mixture of *K. pneumoniae* and *K. quasipneumoniae*, totaling 117 samples across the two species.

### 2.3 MALDI‐TOF MS Analysis

MS analyses were performed on a MALDI Biotyper sirius system (Bruker Daltonics, Germany) using the protocol previously described (Pizzato et al. 2022). The mass spectra were scanned in the range of *m*/*z* 1000–3000. The mass profiles were acquired using FlexControl 3.4 software (Bruker Daltonics, Germany). The spectra were recorded in the linear negative‐ion mode (laser intensity 65%, ion source 1 = 15.00 kV, ion source 2 = 13.70 kV, lens = 4.45 kV, detector voltage = 2698 kV, pulsed ion extraction = 200 ns). Each spectrum corresponded to ion accumulation of 2000–5000 laser shots randomly distributed on the spot. The spectra obtained were processed with default parameters using FlexAnalysis v.3.4 software (Bruker Daltonics, Germany).

### 2.4 Pre‐Processing of Lipid Spectra Data

The bioinformatics analysis pipeline used R version 4.1.2. The method described here used code adapted from a study by Gibb and Strimmer (2015). “MALDIquant” (version 1.21) and “MALDIquantForeign” (version 0.13) packages were used to pre‐process the spectra data for all *K. pneumoniae* and *K. quasipneumoniae* samples. First, a square root transformation (sqrt) was performed on the intensities of the spectra. The intensity values were then smoothed using the Savitzky–Golay method. The baseline of the mass spectrometry data was estimated and then removed using the Statistics‐sensitive Nonlinear Iterative Peak‐clipping algorithm (SNIP). Intensity values were normalized using the Total Ion Current (TIC) method, and then spectra were aligned. A signal‐to‐noise ratio of 3 (SNR = 3) and a half window size of 20 (HWS = 20) were used to detect peaks above the defined threshold in the mass spectrometry data. Following this, the peak binning function was used to look for similar peaks across different spectra and equalize their mass. Finally, peaks that occurred infrequently within a same species group were removed from the data. After this pre‐processing, the result was a two‐dimensional feature matrix containing peak intensity information for the spectra of all samples.

### 2.5 Machine Learning

The approach used was in agreement as the one previously described (Pizzato et al. 2022). Briefly, after above pre‐processing workflow, the feature matrix was converted into both a naïve binary absence–presence matrix (replaced non‐negative and missing value with 1 and 0 respectively in feature matrix, true labels were not utilized.) and a dichotomized binary matrix. (For each feature [*m*/*z*], a threshold is determined by considering true labels. Intensities above that threshold will be set to 1, otherwise 0, via R packages “binda” [version 1.0.4]; Gibb and Strimmer 2015.) Hierarchical clustering was applied on a naïve binary feature matrix to figure out if different species can be separated in an unsupervised manner.

Binary discriminant analysis was then applied to the dichotomized binary feature matrix to identify and rank the most differentially expressed peaks across the spectra and ascertain whether any of these peaks from the lipid profiles could be used for class prediction, that is, to determine whether the spectra belonged to a sample of *K. pneumoniae* or *K. quasipneumoniae* (by computing *t*‐scores between the group means in each species). The top‐ranked peaks were used to test their class prediction ability. Data were further split into training and testing data (randomly picked 70% of all samples for training and rest for testing) to study the robustness of the top‐ranked features in terms of classification of the two species.

## 3 RESULTS

### 3.1 *K. pneumoniae* and *K. quasipneumoniae* Showed Distinct Lipid Profile

To evaluate lipid profiling for discriminating *K. pneumoniae* from *K. quasipneumoniae* using a standard MALDI Biotyper Sirius system, we analyzed a panel of 20 *K. pneumoniae* and 19 *K. quasipneumoniae* clinical isolates, with each sample processed in technical triplicate. The samples were prepared for membrane lipid enrichment, and mass spectra were acquired in linear negative ion mode. The *m/z* 800–2300 range was selected for analysis based on optimal signal‐to‐noise ratio (S/N > 3) and mass resolution (> 200), ensuring suitability for subsequent data processing.

**Figure 1:**
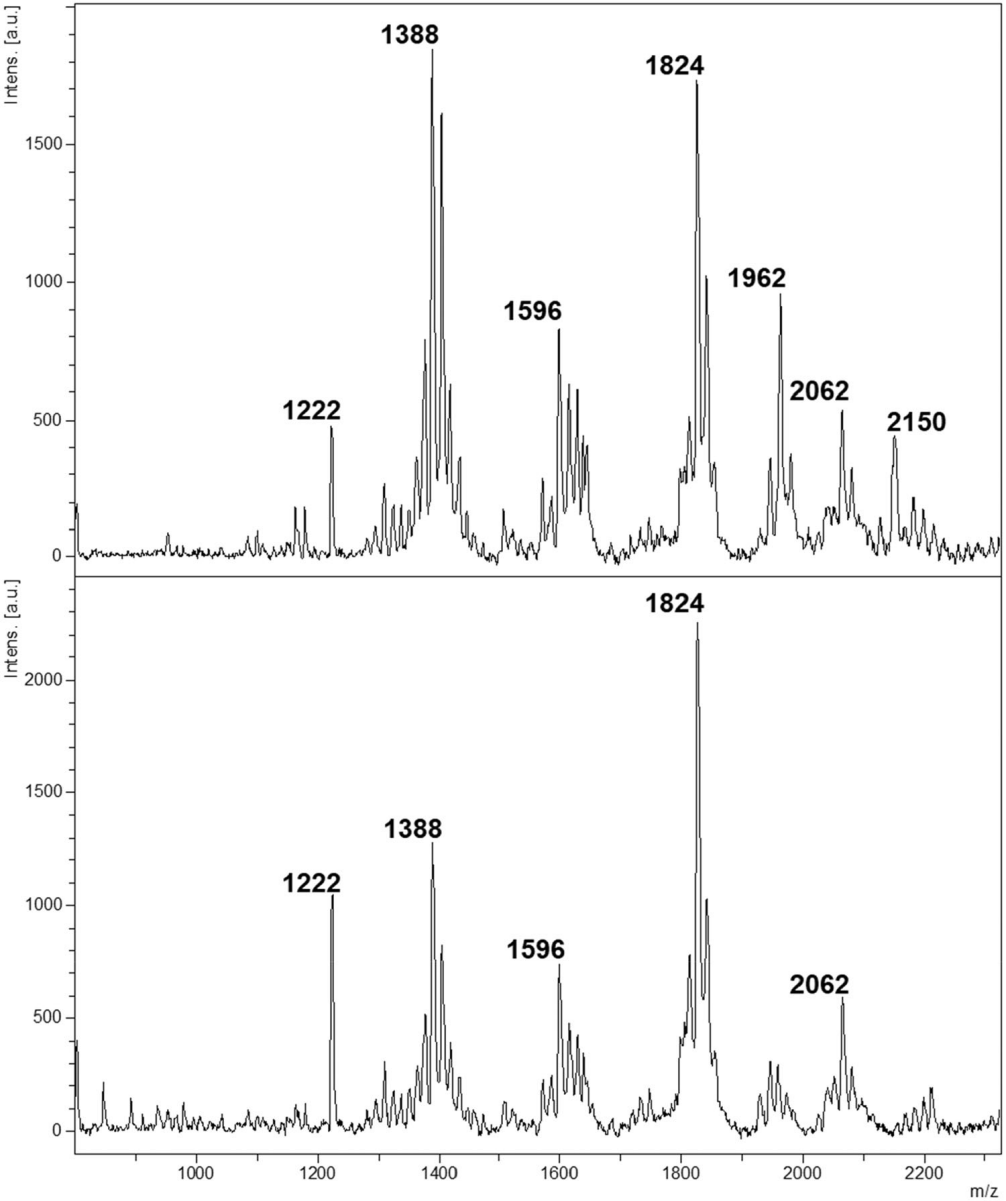
Linear negative ion mode mass spectra of *K. pneumoniae* (top panel) and *K. quasipneumoniae* (bottom panel).

A total of 117 spectra (60 from *K. pneumoniae* [20 isolates × 3 replicates]; 57 from *K. quasipneumoniae* [19 isolates × 3 replicates]) were pre‐processed before further data analysis. As shown in Figure 1, spectra from both species displayed prominent peaks centered at *m/z* 1824, identified as bis‐phosphorylated, hexa‐acylated lipid A. Additional common peaks at *m/z* 1388 (identified as cardiolipin) and *m/z* 2062 (identified as bis‐phosphorylated, hepta‐acylated lipid A, resulting from palmitoylation at the C‐1 acyl‐oxo‐acyl position of the *m/z* 1824 molecule) indicated shared lipid features.

Despite these similarities, peak intensities at *m/z* 1388 and *m/z* 2062 differed between species. Furthermore, the peak at *m/z* 1962 demonstrated higher abundance in *K. pneumoniae*. To determine whether lipid profiles could effectively differentiate the species, we employed machine learning methodologies. This analysis targeted the identification of specific differential markers capable of reliably distinguishing *K. pneumoniae* and *K. quasipneumoniae*.

### 3.2 Machine Learning Allows Discrimination of *K. pneumoniae* and *K. quasipneumoniae* Clinical Isolates

**Figure 2:**
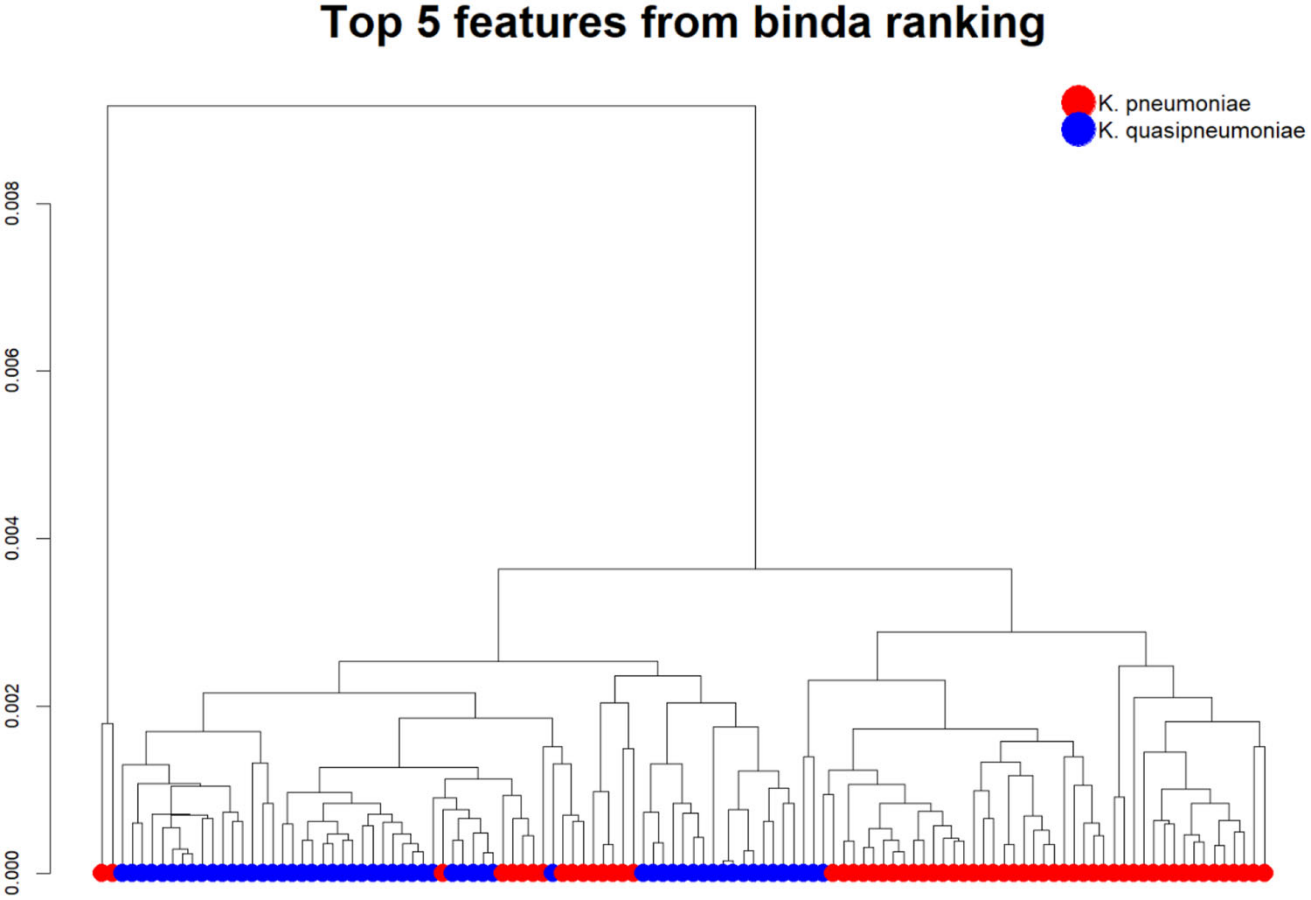
Dendrograms showing hierarchical clustering of *K. pneumoniae* and *K. quasipneumoniae*. Red indicates *K. pneumoniae* isolates and blue indicates *K. quasipneumoniae*. Clustering of species was performed using the naïve binary absence presence matrix.

Following established data pre‐processing protocols (Tang et al. 2019), initial analysis of the lipid profiles revealed unsupervised clustering of the top five ranked features (Figure 2). Subsequently, these top‐ranked peaks were identified and their robustness for classification was validated.

**Figure 3:**
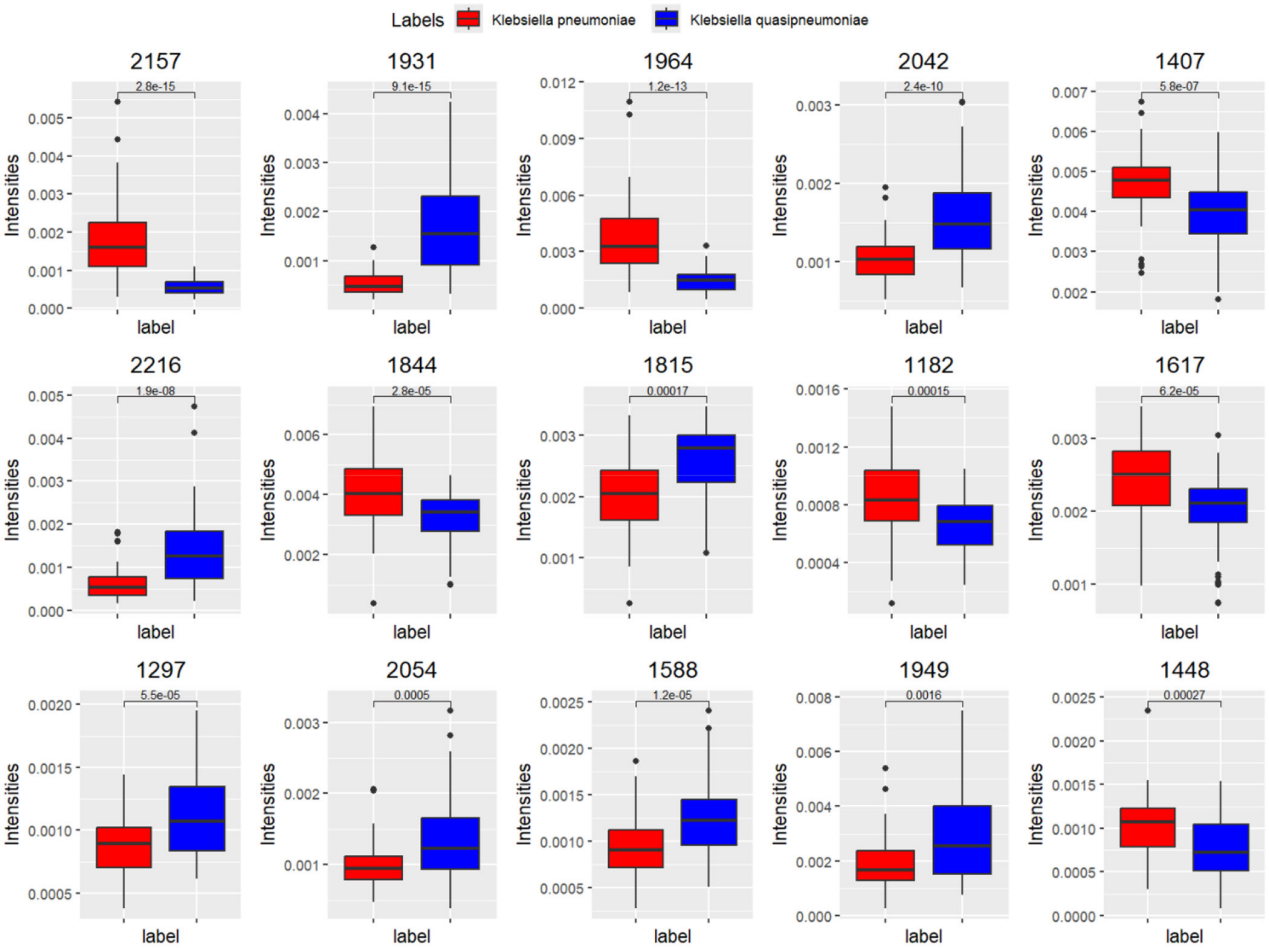
Top 15 ranked features reported from binda (ranked from left to right, from top to bottom). *p* values were calculated from *t*‐tests.

Using the dichotomized matrix in a supervised approach, we extracted the top‐ranked peaks (Table S2) via multi‐class discriminant analysis employing binary predictors (Gibb and Strimmer 2015). Using the “binda” R package, the intensities of the 15 top‐ranked peaks efficiently distinguished *K. pneumoniae* and *K. quasipneumoniae* (Figure 3).

### 3.3 Validation of the Robustness of Top‐Ranked Peaks via Supervised Learning and Using Randomly Selected Features as Control

**Figure 4:**
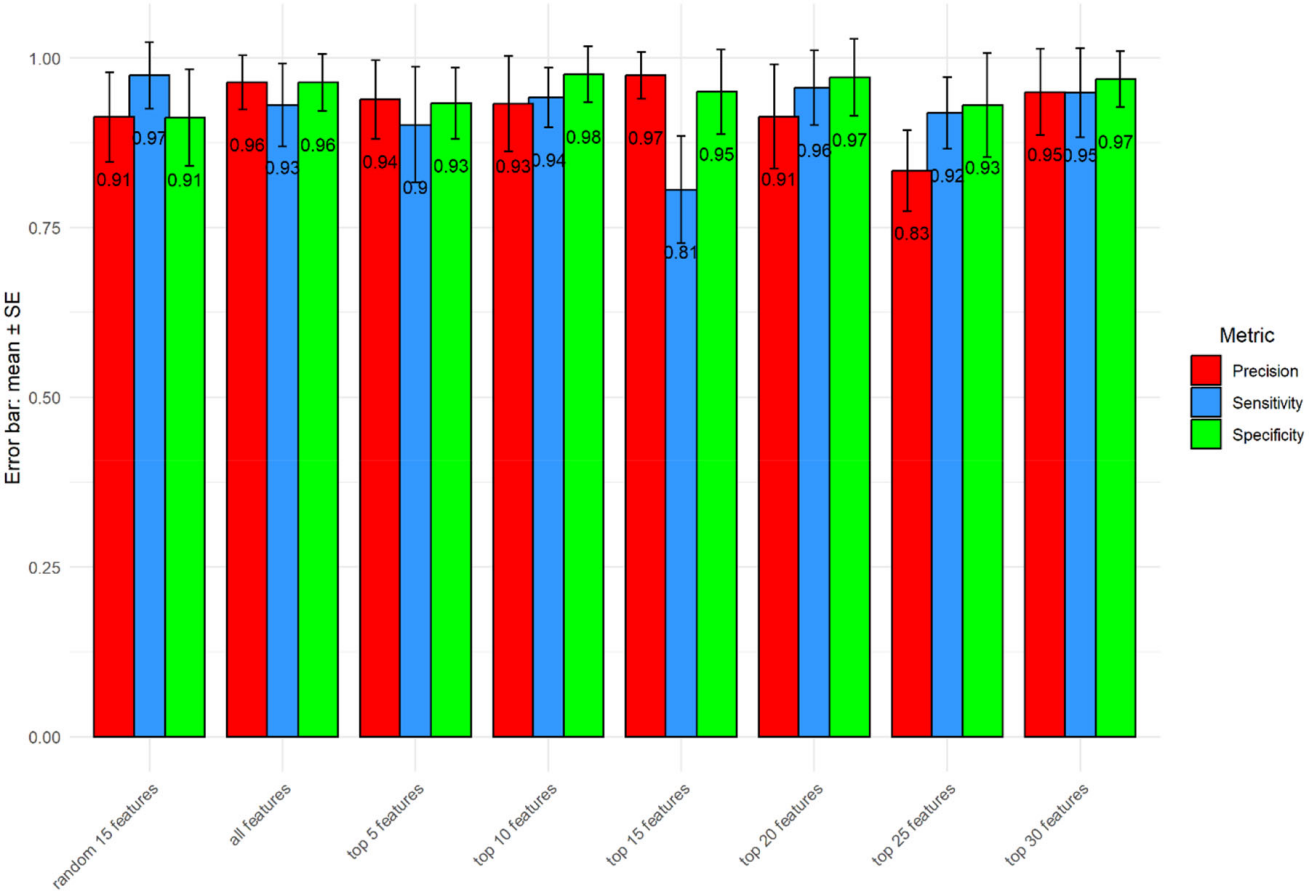
Classification metrics including precision, sensitivity, and specificity.

**Figure 5:**
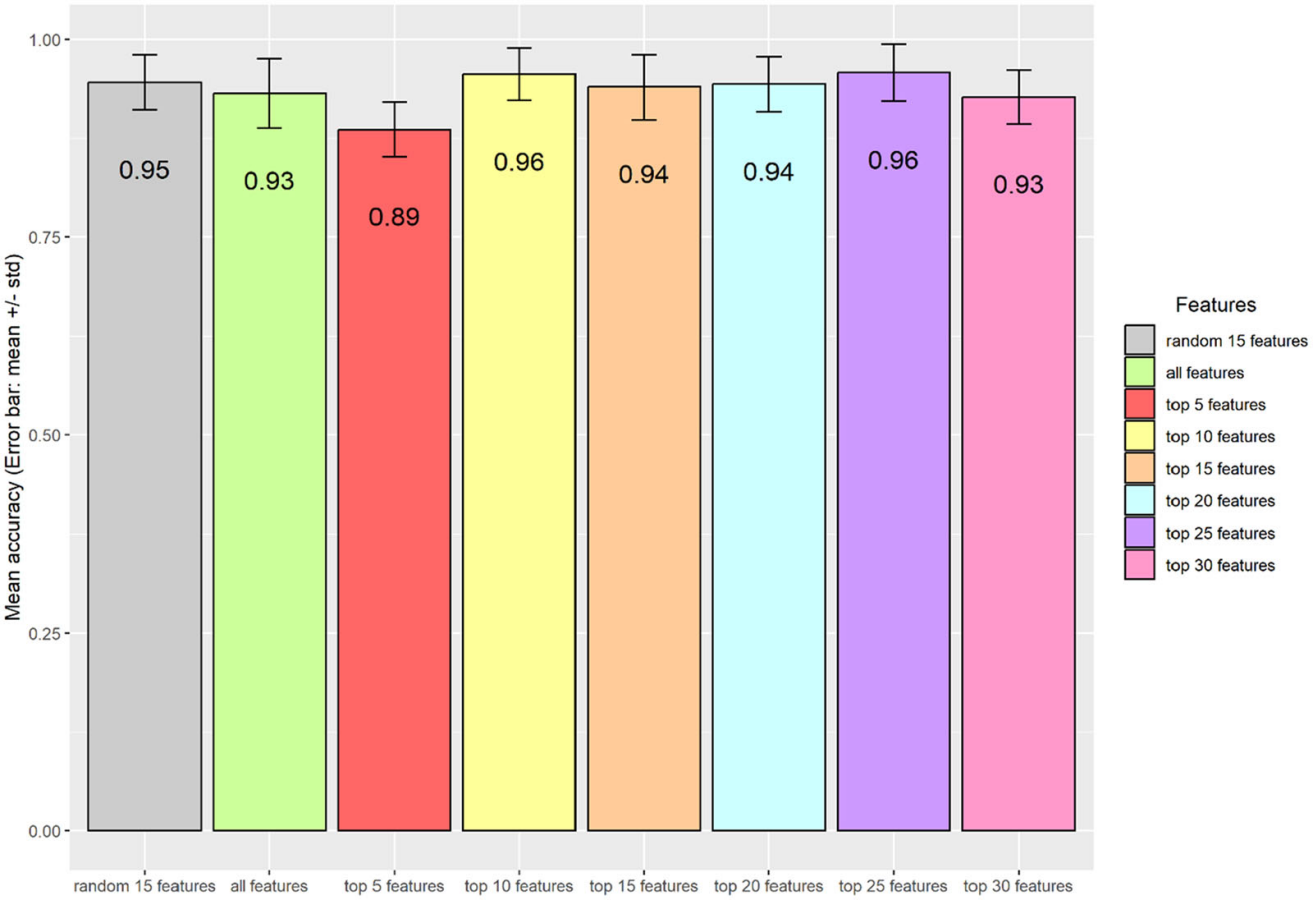
Bar plots showing accuracy values for species prediction (between *K. pneumoniae* and *K. quasipneumoniae*) using top‐ranked features; all features and randomly selected features. The accuracy values come from the analysis being repeated 100 times of splitting training and testing data and randomly selection of peaks for control.

To validate the classification robustness of the top‐ranked peaks for distinguishing the two bacterial species, we partitioned the data set into training (70% of 117 samples) and testing subsets. The random separation of training and testing data were repeated 100 times. Various numbers of top‐ranked peaks, either all peaks (*n* = 44) or a subset of randomly selected peaks (*n* = 15), were used as control. Consistent with a previous report from Tang et al. (2019), minimal differences in accuracy rates were observed between the groups using different controls. This finding supports previous reports that only a subset of top‐ranked peaks is sufficient for classification (Gibb and Strimmer 2015; Conrad et al. 2017). Using top‐ranked peaks, we were able to achieve relatively higher performance metrics, in terms of precision, sensitivity, and specificity for identifying *K. pneumoniae* and *K. quasipneumoniae* (Figure 4). In addition, the accuracy rates, based on comparison between *K. pneumoniae* and *K. quasipneumoniae,* enable discrimination of the two species, which is consistent with the data shown in Figure 5.

Overall, the five top‐ranked peaks are sufficient for discriminating the species (*m/z* 2157, *m/z* 1931, *m/z* 1964, *m/z* 2042, *m/z* 1407) (Table S2).

## 4 DISCUSSION

MALDI‐TOF MS is widely applied in clinical microbiology for identification of microorganisms (Singhal et al. 2015; Kostrzewa et al. 2019). Although capsule genotyping and conventional protein‐based MALDI‐TOF MS are commonly used for *Klebsiella* species identification, their diagnostic accuracy is limited by the high genetic similarities among these closely related strains (Long et al. 2017). This genomic similarity can be quantified using metrics such as average nucleotide identity (ANI). ANI values above 95%–96% generally indicate the same species, while values between 93% and 95% suggest very closely related but distinct species. The primary focus of this study, *K. pneumoniae* and *K. quasipneumoniae*, have an ANI value around 93%–94%, indicating their high similarity and the difficulties in species identification (Brisse et al. 2014). In this project, the negative ion mode of MALDI‐TOF MS was used to identify distinctive features in the lipid profiles of *K. pneumoniae* and *K. quasipneumoniae*, which has the potential as a novel species classification method.

To differentiate these closely related *Klebsiella* species, this study focuses on identifying discriminative features between *K. pneumoniae* and *K. quasipneumoniae*. Methodological refinements such as testing alternative mass spectrometry matrices or optimizing preprocessing parameters could enhance result accuracy and reproducibility. These adjustments could further enhance the accuracy and reproducibility of the results. While Monte‐Carlo cross‐validation was used to test the mean accuracy of the predictions from different rankings, other cross‐validation methods, such as *k*‐fold cross‐validation, could also be used to reduce variability between results.

Based on our data, further investigations should focus on identifying the top‐ranked molecules in our supervised studies. For example, techniques such as tandem mass spectrometry (MS/MS) can be used to reveal the biomolecular identities underlying those top‐ranked peaks.

The potential clinical implications of lipid profile‐based microbial identification are substantial. The rapid and accurate classification of closely related *Klebsiella* species can distinguish their different clinical pathogenicity, epidemiology, and potential antibiotic resistance, hence improving patient care with timely and appropriate treatments (Watanabe et al. 2022). Although WGS is known for its high accuracy in species identification, its sample preparation process is labor‐intensive and time‐costing because most WGS is done outside of diagnostic laboratories. Thus, rapid diagnostic methods based on already existing equipment within clinical setting are more practical. The development of diagnostic protocols incorporating these lipid profile‐based biomarkers, identified in this study, could reduce the time to diagnosis and improve the management of infections caused by *Klebsiella* species. Clinical implementation of these findings requires addressing practical and regulatory considerations. Standardized protocols that ensure reproducibility must be developed, alongside evaluations of cost‐effectiveness and scalability for routine negative‐ion mode MALDI‐TOF MS diagnostics.

In conclusion, while negative‐ion mode MALDI‐TOF MS lipid profiling shows initial promise for distinguishing *K. pneumoniae* from *K. quasipneumoniae*, expanded validation using diverse geographical and clinical isolates remains essential. Continued research in this area holds the potential to develop more rapid, accurate, and cost‐efficient diagnostic tools for clinical microbiology, ultimately improving the identification and treatment of infections caused by closely related *Klebsiella* species. By expanding the scope of this study and addressing practical considerations, more accurate and rapid microbial diagnostics can be achieved, benefiting patient outcomes and public health.

## Author Contributions

**Mari Nishikawa:** data curation, investigation, methodology. **Wenhao Tang:** data curation, methodology, investigation, software. **Markus Kostrzewa:** resources, writing – review and editing. **Jonah Rodgus:** investigation, data curation, writing – review and editing. **Frances Davies:** resources, writing – review and editing. **Yi Liu:** writing – review and editing. **Elita Jauneikaite:** resources, writing – review and editing, conceptualization, data curation, supervision, investigation, methodology. **Gerald Larrouy‐Maumus:** conceptualization, investigation, funding acquisition, writing – original draft, supervision, data curation, methodology.

## Ethics Statement

The isolates were collected in accordance with ethics reference 21/LO/0170 (279677), protocol 21HH6538 Investigation of epidemiological and pathogenic factors associated with infectious diseases.

## Conflicts of Interest

M.K. is an employee of Bruker Daltonics GmbH & Co. KG, the manufacturer of the MALDI‐TOF MS system used in this study, and MBT Lipid Xtract Kit. The other authors declare no conflicts of interest.

## Supporting information

Table S1.

Table S2.

supmat.

## Data Availability

All data are provided in full in the results section of this paper. Whole‐genome sequencing raw data (Illumina reads) for isolates used in this study have been deposited in European Nucleotide Archive (https://www.ebi.ac.uk/ena/browser/home) under BioProjects PRJEB83319 (*K. pneumoniae*) and PRJEB85157 (*K. quasipneumoniae*), see Table S1.
