# Supplementary material for: Discrimination of Klebsiella pneumoniae and Klebsiella quasipneumoniae by MALDI‐TOF Mass Spectrometry Coupled With Machine Learning
Source: Microbiologyopen. 2025 Jul 15;14(4):e70035. doi: 10.1002/mbo3.70035 (PMC12260210; doi:10.1002/mbo3.70035)
Supplement: Supplementary file 3 — supmat. [file MBO3-14-e70035-s001.docx]

**Appendices**

**Table S1: List of strains sued in the study**

**Table S2: List of peaks across dataset**
